# A Feature based Reconstruction Model for Fluorescence Microscopy Image Denoising

**DOI:** 10.1038/s41598-019-43973-2

**Published:** 2019-05-22

**Authors:** Suman Kumar Maji, Hussein Yahia

**Affiliations:** 1 Department of Computer Science & Engineering, Indian Institute of Technology Patna, Patna, 800 013 India; 2 Geostat, INRIA Bordeaux Sud-Ouest, 200 rue de la Vieille Tour, 33405 Talence Cedex, France

**Keywords:** Biological techniques, Image processing

## Abstract

The advent of Fluorescence Microscopy over the last few years have dramatically improved the problem of visualization and tracking of specific cellular objects for biological inference. But like any other imaging system, fluorescence microscopy has its own limitations. The resultant images suffer from the effect of noise due to both signal dependent and signal independent factors, thereby limiting the possibility of biological inferencing. Denoising is a class of image processing algorithms that aim to remove noise from acquired images and has gained wide attention in the field of fluorescence microscopy image restoration. In this paper, we propose an image denoising algorithm based on the concept of feature extraction through multifractal decomposition and then estimate a noise free image from the gradients restricted to these features. Experimental results over simulated and real fluorescence microscopy data prove the merit of the proposed approach, both visually and quantitatively.

## Introduction

The ability of fluorescence microscopy to identify and distinguish cells as well as cellular particles, to the extent of a single molecule, has made it an essential tool in biomedical sciences. Its growth is closely linked with the development of synthetic proteins called fluorophores that are designed to target specific cellular objects, and are characterized by their individual fluorescent profile like color, emission and excitation wavelength, etc. Image formation process in fluorescence microscopy can be summarized as follows: Fluorophores introduced to an experimental biological sample (like yeast cell culture) tags themselves to specific cellular objects. When this sample is imaged, using conventional light microscopes, the fluorophores present in the sample start emitting fluorescence. This emitted light is captured by the detector, after filtering it out from the sample excitation light. As a result a high contrast fluorescent image of the target objects are formed on the detector against a black (no light) background, thereby making the specific object of interest visible. The sample data is then recorded, in the form of images, at specific intervals and for a specific duration as per necessity. The resultant image data is then used for further interpretation of the cellular object.

Although beneficial, its primary objective of interpreting from the image data is restricted due to the presence of noise in it. The effect of noise is primarily due to two reasons: (i) Not all photons, emitted by the excited fluorophores, are captured by the detector, and (ii) imperfections in the imaging system contributes to measurement noise. Other than these, due to biological reasons like photo-toxicity, photo-bleaching the excitation time of the sample has to be limited which also results in limited photon emission. All these factors contribute to the formation of a spatially downgraded low signal-to-noise-ratio (SNR) image. The contribution to noise due to loss of photons is generally modeled as a Poisson process while measurement noise is modeled as a Gaussian process. Noise in fluorescence microscopy therefore follows a mixed Poisson-Gaussian statistics.

Image denoising is a class of computational image processing algorithms that aim to restore spatially downgraded acquired images. Recent developments in denoising algorithm for fluorescence microscopy has gained wide attention in the scientific community. A common approach is to model the microscopy image formation process using Bayesian framework, and subsequently solve it using the maximum likelihood and/or the maximum *a posteriori* approach. The authors in^1–3^ have followed this principle, after adapting the general Bayesian model for Poisson processes. The utility of wavelets in designing denoising mechanisms for fluorescent images have also been explored. These methods^4–7^ work on the principle of employing post-processing techniques on wavelet coefficients, and then through an inverse wavelet transform on the processed coefficents, reconstruct a denoised image. In this regard, the SURE-LET approach^6,7^ has proved to be a reliable technique. Here, instead of manipulation, interscale dependencies between the wavelet coefficients are taken into consideration for reconstructing a denoised image. Patch-based denoising techniques^8–10^, where instead of a single pixel blocks of pixel called patches are considered for processing (filtering) the noisy image, are also employed to denoise fluorescent microscopy data. In this regard, the non-local patch based denoising technique proposed in^9^ is quite popular. In^11^, the authors propose an unbiased risk estimator based denoising scheme. Here, the conventional Stein’s unbiased risk estimator^12^ was remodeled to address Poisson-Gaussian noise statistics in fluorescence microscopy. Variance stabilization based denoising methods^13,14^, where authors use Anscombe transform^15^ for stabilizing the mixed Poisson-Gaussian noise behavior to mostly Gaussian noise, are also effective. Both the techniques, commonly known as GAT + BM3D^13^ and VST + BM3D^14^, are extension of the highly popular BM3D denoising algorithm^10^ (developed to address Gaussian noise only).

**Figure 1:**
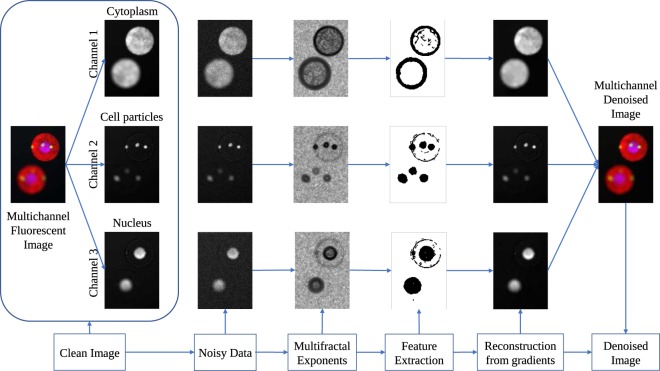
Workflow of the proposed denoising scheme. We consider a simulated multichannel fluorescence microscopy data set as the ground truth (clean image), generated using SIMCEP^24^. Simulated mixed Poisson-Gaussian noise is then added to create the effect of fluorescence microscopy acquisition. Multifractal exponents are then computed over this noisy image and ${ {\mathcal M} }_{\infty }$ extracted. Finally, a denoised image is reconstructed from the information of the gradients restricted to ${ {\mathcal M} }_{\infty }$ (see section Methods for details).

In this paper, we formulate a dual step process for denoising fluorescence microscopy images. The first step involves the computation of multifractal exponents associated to the noisy fluorescent sample. Then through a hierarchical decomposition process of these exponents we obtain a set of pixels, as our features, that encode only the sharp transition in the image and rejects any backgorund noise information. The gradients corresponding to this pixel set are naturally the least corrupted (by noise) gradients of the image, as well as the most informative. Hence reconstruction from these gradients will ensure negligible noise propagation resulting in a denoised image. Step 2, therefore, consists of reconstructing the whole image from the gradients restricted to the features, by solving a discrete Poisson equation with Neumann boundary conditions. The process is summarized using a workflow shown in Fig. 1, where a simulated multichannel fluorescence microscopy image is used for demonstration.

## Methods

### Problem formulation

The image formation process in fluorescence microscopy can be mathematically modelled as follows: If *b* is the observed image and *v* the true image, then we have^11,16^:

1 $$b={g}_{0}z+\varepsilon \,{\rm{with}}\,z\sim {\mathscr{P}}(v/{g}_{0})\,{\rm{and}}\,\varepsilon \sim {\mathscr{N}}(b,{\sigma }^{2})$$

where *z* is the number of photons collected by the detector and is generally modelled as a Poisson process. *g*_0_ is the gain of the sensor detector. $\varepsilon$ takes into account the measurement noise and is modeled as additive Gaussian noise with given variance *σ*^2^. Values of *g*_0_, $\sigma \ge 0$ control the level of Poisson and Gaussian noise, respectively, in the signal.

Our objective is to find a restored image $\hat{v}$ from *b* such that $\hat{v}\simeq v$. This is done in two steps. In first step we extract multifractal features from the observed image *b*. In the second step we reconstruct a denoised image $\hat{v}$ from the gradients of *b* restricted to the features. The process can be visualized from Fig. 1, the mathematical modeling being explained in the successive subsections.

### Multifractal decomposition and feature extraction

Multifractal systems came into existence for their ability to define irregular structures and chaotic systems. They work on the principle of self-similarity in objects, and are statistically employed to exploit redundancy (or patterns) in objects at multiple scales. Multifractal systems are characterized by values known as multifractal exponents, denoted by *d*(*x*), that are naturally resistent to noise. The hierarchical organization of a multifractal system can be exploited through a decomposition process associated to its multifractal exponents, which leads to their underlying fractal components (or sets)^17–21^. We denote these sets by ${ {\mathcal M} }_{d}$. Of these sets, there exist a specific set ${ {\mathcal M} }_{\infty }$ which encodes only those pixels that are related to sharp transitions within an image and whose visual appearence can be related to an edge representation of an image^20–22^. Our objective is to compute the set ${ {\mathcal M} }_{\infty }$ for the observed image *b*, so that we are able to extract the most informative features in the image and get rid of the noisy pixels.

We therefore model the observed image *b* accordingly. Let $\mu (x,r)$ be a positive measure of *b*, defined for every pixel $x\in {\rm{\Omega }}$. $\mu (x,r)$ is obtained after convolving *b*(*x*) with a certain scale dependent operator ${\mathbb{H}}(x,r)$ i.e., $\mu (x,r)={\mathbb{H}}(x,r)\otimes b(x)$ and $\otimes$ denote the convolution operator. Multifractal analysis states that the wavelet projections scale as power laws in *r*^19,22^ and are therefore prime candidates of choice for the scale-dependent operator ${\mathbb{H}}(x,r)$. The chosen measure $\mu (x,r)$, henceforth denoted by ${\mu }_{{\rm{\Psi }}}(x,r)$, is obtained after convolution of *b*(*x*) with wavelets from the family ${\rm{\Psi }}(x)=\frac{1}{{(1+|x{|}^{2})}^{\gamma }}\,({\rm{for}}\,\gamma =1,2,3,4)$ and averaging the resulting coefficients. We use a microcanonical multifractal evaluation^19,20^, where the measure ${\mu }_{{\rm{\Psi }}}(x,r)$ for any point $\overrightarrow{x}\in {\rm{\Omega }}$ satisfies the following equation:

2 $${\mu }_{{\rm{\Psi }}}(x,r)\approx {\alpha }_{{\rm{\Psi }}}(x){r}^{d(x)}\,(r\to 0)$$

where ${\alpha }_{{\rm{\Psi }}}(x)$ is a constant which depends on the wavelet ${\rm{\Psi }}$ (independent of the scale *r*) and is calculated as the average value of the wavelet projection over the whole signal. The exponent *d*(*x*) is called the multifractal exponent or the singularity exponent. For small number of *r*’s, the above equation satisfies the equality criteria. Taking log on both sides, we get:

3 $$\mathrm{log}({\mu }_{{\rm{\Psi }}}(x,r))=\,\mathrm{log}({\alpha }_{{\rm{\Psi }}}(x))+d(x)\,\mathrm{log}(r)\,(r\to 0),$$

from which *d*(*x*) can be obtained by a linear regression of $\mathrm{log}({\mu }_{{\rm{\Psi }}}(x,r))$ vs. log(*r*). The exponents *d*(*x*), which is an image of the same size of *b*(*x*), describes the local irregularities at each pixel *x*. The fractal components ${ {\mathcal M} }_{d}$ are level sets of the function *d*(*x*) and are defined as:

4 $${ {\mathcal M} }_{d}=\{x\,{\rm{s}}.\,{\rm{t}}.\,d(x)=d\}$$

The set ${ {\mathcal M} }_{\infty }$ consists of the components associated with the smallest possible value ${d}_{\infty }$ and can be interpreted as:

5 $${ {\mathcal M} }_{\infty }=\{x\,{\rm{s}}.\,{\rm{t}}.\,d(x)={d}_{\infty }=\,{\rm{\min }}(d(x))\}$$

Visual observation of this set reveals a feature set that has resemblance to an edge map of the image. When applied over noisy samples, ${ {\mathcal M} }_{\infty }$ maintains its stability over extracting the image features while at the same time eliminating the backgorund noise. This can be observed for the case of the simulated multichannel fluorescent microscopy data shown in Fig. 1. ${ {\mathcal M} }_{\infty }$ therefore retains the most informative features of the image and can be interpreted as the most informative set. The procedure for calculating the ${ {\mathcal M} }_{\infty }$

**Algorithm 1:**
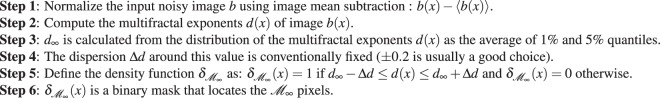
Determining $${ {\mathcal M} }_{\infty }$$.

pixels is presented in algorithm 1.

### Image reconstruction from gradients

After being able to extract features from the noisy image *b*(*x*), in the form of ${ {\mathcal M} }_{\infty }$, we now intend to reconstruct the whole image from this feature set. We therefore propose to reconstruct an image, for every point *x* in the image domain, from the gradient $\nabla b$ evaluated over ${ {\mathcal M} }_{\infty }$. We say ${\delta }_{{ {\mathcal M} }_{\infty }}$ to be the standard density measure restricted to the set ${ {\mathcal M} }_{\infty }$ and *b*_*x*_, *b*_*y*_ to be the gradients of the noisy image. So, essentially we are trying to estimate a surface from ${\hat{b}}_{x}=\nabla {b}_{x}\cdot {\delta }_{{ {\mathcal M} }_{\infty }}$ and ${\hat{b}}_{y}=\nabla {b}_{y}\cdot {\delta }_{{ {\mathcal M} }_{\infty }}$, where · denotes the dot product. Since ${\hat{b}}_{x},{\hat{b}}_{y}$ are the gradients corresponding to the most informative set ${ {\mathcal M} }_{\infty }$, they are therefore the most informative and least corrupted (by noise) gradients of the image. The surface estimated from these gradients will therefore be a denoised image.

Let *v*_*x*_, *v*_*y*_ be the gradients of the clean image *v*. The goal will be to minimize the following energy equation:

6 $$\mathop{{\rm{argmin}}\,}\limits_{{\rm{v}}}\,\iint ({({v}_{x}-{\hat{b}}_{x})}^{2}+{({v}_{y}-{\hat{b}}_{y})}^{2})dxdy$$

The associated Euler-Lagrange equation gives the Poisson equation:

7 $${\rm{div}}({v}_{x},{v}_{y})={\rm{div}}({\hat{b}}_{x},{\hat{b}}_{y})$$

where ‘div’ refers to the divergence operator and is defined as ${\rm{div}}\,({\hat{b}}_{x},{\hat{b}}_{y})=\frac{\partial {\hat{b}}_{x}}{\partial x}+\frac{\partial {\hat{b}}_{y}}{\partial y}$ and $\nabla$ denotes the gradient operator. Equation (7) can be written in the form of a diffusion equation^23^:

8 $${\rm{div}}(J[\begin{array}{l}{v}_{x}\\ {v}_{y}\end{array}])={\rm{div}}(J[\begin{array}{l}{\hat{b}}_{x}\\ {\hat{b}}_{y}\end{array}])$$

where *J* is a 2 × 2 symmetric, positive definite diffusion tensor matrix with elements $({j}_{11}\,{j}_{12},{j}_{21}\,{j}_{22})$. Equation (8) then takes the form:

9 $${\rm{div}}\mathop{\underbrace{([\begin{array}{ll}{j}_{11} & {j}_{12}\\ {j}_{21} & {j}_{22}\end{array}]\cdot [\begin{array}{l}{v}_{x}\\ {v}_{y}\end{array}])}}\limits_{{{\rm{\Delta }}}_{j}v}=\mathop{\underbrace{{\rm{div}}([\begin{array}{ll}{j}_{11} & {j}_{12}\\ {j}_{21} & {j}_{22}\end{array}]\cdot [\begin{array}{l}{\hat{b}}_{x}\\ {\hat{b}}_{y}\end{array}])}}\limits_{{b}_{j}}$$

with ${j}_{12}={j}_{21}$ due to symmetry. The term on the right-hand side is a known quantity. Δ_*j*_ is the weighted Laplacian matrix whose weights are determined by the elements of *J*. The solution is given by $\hat{v}={{\rm{\Delta }}}_{j}^{-1}{b}_{j}$, where $\hat{v}$ is an estimate of *v* and our desired denoised image. The results are shown in section Results. Computation process of the elements of matrix *J* is summarized in algorithm 2.

**Algorithm 2:**
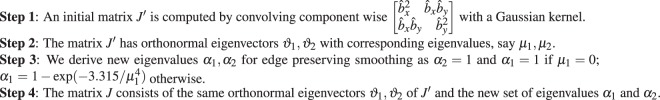
Determining the matrix *J*.

## Results

Two types of data have been used for experimental validation of the proposed denoising scheme: simulated fluorescence microscopy data and real fluorescence microscopy acquisition data. The competing state-of-the-art denoising algorithms that has been used for experimental validation purposes (discussed extensively in Introduction) are the following:

- **SURE-LET** - Wavelet based Image Denoising^7^.
- **VST + BM3D** - Variance Stabilization based Iterative Denoising^14^.
- **GAT + BM3D** - Optimal Inversion of Anscombe Transform based Denoising^13^.
- **PG-URE** - An Unbiased Risk Estimator for Image Denoising^11^.

### Simulated data

**Figure 2:**
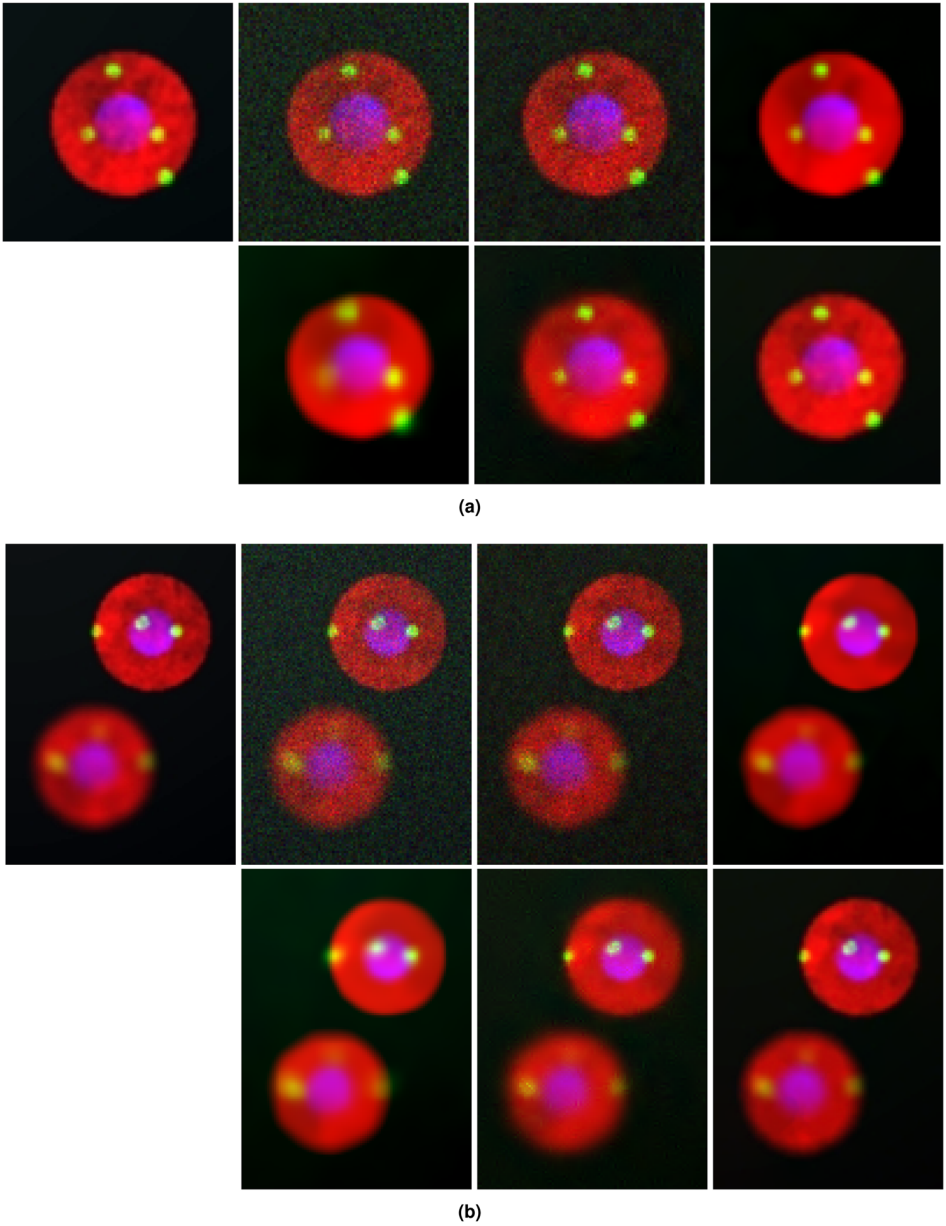
Application of different denoising algorithms over multichannel simulated fluorescence microscopy data generated using SIMCEP^24^. From left to right: Excerpt of simulated multichannel image as ground truth, after adding mixed Poisson-Gaussian noise using equation (1), denoised outputs of SURE-LET, VST + BM3D, GAT + BM3D, PG-URE and Proposed scheme.We demonstrate results on two different excerpts from the simulated image. (**a**) Single cell excerpt and (**b**) multiple cell excerpt.

The simulated data sets that we use for our experiment is generated using SIMCEP^24^, a computational framework for simulating fluorescent microscopy cell populations. The images that we use have three fluorescently labeled channels: the cytoplasm (in red), the nucleus (in blue) and cell particles (in yellow). The images are simulated with the following parameters: image size = 512 × 512 pixels, amount of cells = 20, number of clusters = 3 and number of subcellular objects = 4. We have simulated five such data (image) sets, as is shown in Fig. S1 of Supplementary Information. For demonstration purpose in the manuscript, we have chosen Data 1 as reference the excerpts of which are shown in Fig. 2. This image serves as our ground truth or clean image. From this image, we generate noisy sample using the image formation model explained in equation (1). The values of *g*_0_, *σ* determines the level of noise in a fluorescent sample. We have performed experiments over five different levels of Poisson-Gaussian noise (${g}_{0},\sigma ={10}^{-3},{10}^{-2.5},{10}^{-2},{10}^{-1.5},{10}^{-1}$), with ${g}_{0},\sigma ={10}^{-3}$ being low noise and ${g}_{0},\sigma ={10}^{-1}$ being high noise^11^.

Denoising is performed over this noisy sample and the results of excerpts are shown in Fig. 2. The results correspond to high noise level with ${g}_{0},\sigma ={10}^{-1}$. SURE-LET shows limited noise removal capability. VST + BM3D and GAT + BM3D are able to remove background noise but at the same time introduces blur in the samples, thereby resulting in loss of details, which is observed more in GAT + BM3D. Diffusion of colors is also observed along the cell boundary. PG-URE performs better than the previous two algorithms in terms of reduced blur and better preservation of details, but suffers from the effect of artefacts as well as diffusion of colors. The proposed technique performs best in terms of denoising compared to the other techniques. Background noise is mostly eliminated, details well preserved and no diffusion of colors and artefacts are observed.

We further justify the visual observation over this high noise scenario of ${g}_{0},\sigma ={10}^{-1}$ through quantitative analysis. We compare the statistics of the residual error, between the clean image and the denoised sample, in terms of the power spectral density (PSD). The procedure is as follows:

- Compute the residual error Δ$v=v-\hat{v}$.
- The PSD is given by the square magnitude of the Fourier Transform of Δ*v* i.e., $| {\mathcal F} ({\rm{\Delta }}v){|}^{2}$.
- Plot the PSD against spatial frequency in the log-log scale.

**Figure 3:**
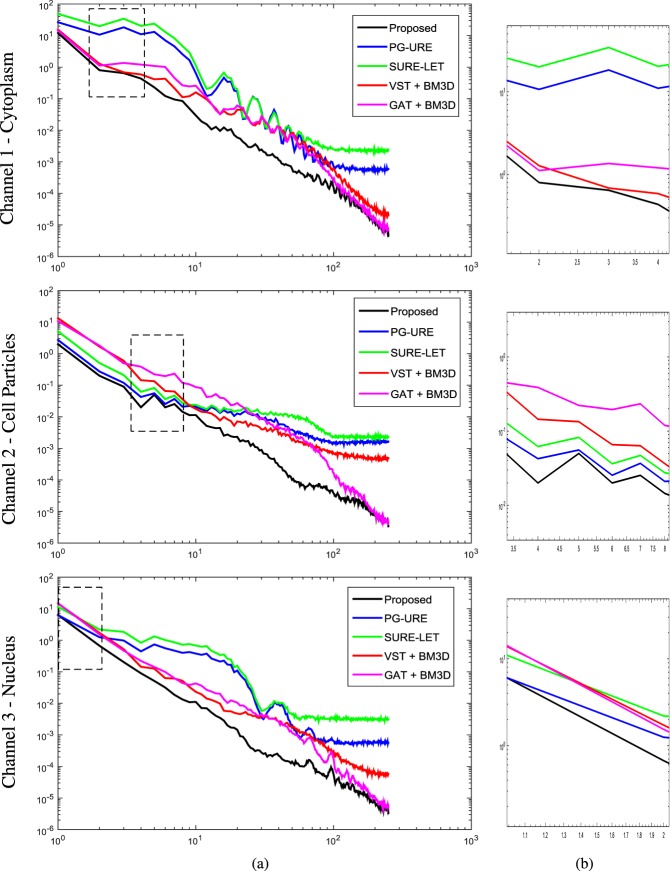
Quantitative analysis of denoising results (shown in Fig. 2) over simulated fluorescence microscopy data set, in terms of residual power spectral density (PSD). (**a**) The residual PSD’s are computed as discussed in section Results, and plotted against spatial frequency in the log-log scale. (**b**) Excerpt corresponding to the black dotted box in (**a**).

The PSD of Δ*v* is computed for all the denoising algorithms, for the three channels, and the results are shown in Fig. 3. Lower the residual error (i.e. better denoising), lower is the curve in the hierarchy. For all the channels it is observed that the PSD curve of the proposed scheme (shown in black solid line) has the least error. Similar result is also observed for the remaining 4 data sets, as can be seen in Fig. S2 of Supplementary Information, which quantitatively validates the superiority of the proposed technique over the other algorithms.

**Figure 4:**
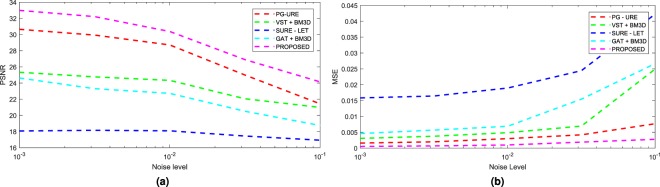
Average values of (**a**) PSNR and (**b**) MSE, over 5 different simulated fluorescent datasets, under different noise variations.

We further validate the denoising performance of all the algorithms, over all the data sets, in terms of the mean square error (MSE) and peak-signal-to-noise ratio (PSNR, expressed in dB) metric governed by the equations:

10 $${\rm{MSE}}=\frac{1}{m\times n}\,\sum _{i,j}\,|v({\overrightarrow{x}}_{i,j})-\hat{v}({\overrightarrow{x}}_{i,j}){|}^{2}$$

11 $${\rm{PSNR}}=20.0\times {\mathrm{log}}_{10}\frac{{\rm{\max }}(v(\overrightarrow{x}))}{\sqrt{{\rm{MSE}}}}$$

where $\hat{v}(\overrightarrow{x})$ represents the denoised image and *m* × *n* the size of the true image $v(\overrightarrow{x})$. Experiments have been performed for the five different noise levels and the results are shown in Tables S1 and S2 of Supplementary Information. The average values of MSE and PSNR, over the 5 data sets, are presented in a graphical format in Fig. 4. We can see that the proposed technique gives the best results over the other denoising techniques, which justifies the results of Fig. 3.

### Fluorescence microscopy data

**Figure 5:**
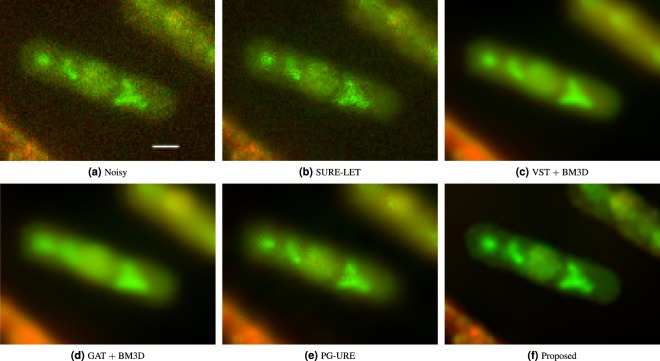
Application of different denoising algorithms over fluorescence microscopy data of biological specimen (yeast). (**a**) Excerpt from multichannel image of experiment 391 from YRC Public Image Repository^25^, (**b**) denoising result of SURE-LET algorithm, (**c**) VST + BM3D algorithm, (**d**) GAT + BM3D algorithm, (**e**) PG-URE algorithm and (**f**) the denoised output of the proposed denoising scheme. The scalebar corresponds to 2 *μm*.

**Figure 6:**
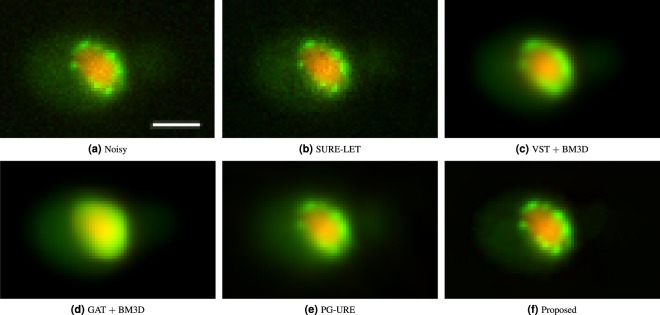
Application of different denoising algorithms over fluorescence microscopy data of biological specimen (yeast). (**a**) Excerpt from multichannel image of experiment 617 from YRC Public Image Repository^25^, (**b**) denoising result of SURE-LET algorithm, (**c**) VST + BM3D algorithm, (**d**) GAT + BM3D algorithm, (**e**) PG-URE algorithm and (**f**) the denoised output of the proposed denoising scheme. The scalebar corresponds to 2 *μm*.

After validating the potential of the proposed scheme over simulated fluorescence microscopy data, we move on to check its performance on real fluorescence microscopy acquisition data. We collect two datasets from the Yeast Resource Centre Public Image Repository (YRCPIR)^25^: experiment 391 and experiment 617. The datasets are the observation image of *Schizosaccharomyces pombe* and *Saccharomyces cerevisiae*, both (yeast) cells, with a 100X objective. The images are of dimension 512 × 512 pixels, with each pixel being of size 0.13 *μm* × 0.13 *μm*. The actual data is composed of fluorescence microscopy acquisitions as well as DIC acquisitions. For our experiments we only consider the fluorescence microscopy acquisition channels. These channels (red and green wavelength channels) show sufficient presence of noise (as can be observed in Figs 5(a) and 6(a)) and is hence suitable for our experimental purpose. Denoising is perfomed on both wavelength channels (red and green) separately and results of excerpts are presented for demonstration.

#### Experiment 391

This data is the fluorescent microscopy acquisition of *Schizosaccharomyces pombe* cells. The actual data is composed of three stacks of images corresponding to three emission wavelengths: red, green and blue. The blue wavelength channel, which is the DIC channel, is not considered for our experiments. Denoising results are shown in Fig. 5.

The image shown in Fig. 5(a) is an excerpt from experiment 391, and shows a single cell with sub-cellular structures. The image shows significant presence of noise. Figure 5(b) shows the denoised output of SURE-LET algorithm^7^. We can observe that noise is eliminated to a certain extent, but not completely. Rather noise suppression has introduced artefacts which has degraded the image quality. Figure 5(c) shows the denoised output of VST + BM3D^14^ and GAT + BM3D^13^ respectively. Noise suppression in both these algorithms is relatively better compared to SURE-LET, but this comes at the cost of high level of blur and diffusion of colors along the boundary of the cell structure. The result of VST + BM3D is, however, relatively better. The result of PG-URE algorithm^11^ is shown in Fig. 5(e). Although there is an improvement in resolution compared to the previous two algorithms, the presence of artefacts is also observed. In Fig. 5(f), we show the denoising results of the proposed technique. Visual inspection clearly justifies its superior performance over the others. We can see that background noise is mostly eliminated and there is low diffusion of colors or artefacts. The effect of blurring barely exists and image sharpness is improved considerably compared to the noisy experimental observation image of Fig. 5(a).

#### Experiment 617

The data here is the fluorescent microscopy acquisition of *Saccharomyces cerevisiae* cells. Here also, the actual data is composed of three stacks of images corresponding to red, green and blue emission wavelengths. We discard the blue DIC channel. The results of denoising are shown in Fig. 6.

In Fig. 6(a), we show a single cell excerpt of the noisy experimental data. The observation is the same as that seen for experiment 391. The proposed denoising scheme achieves clear visible superiority over the other algorithms, with minimal background noise, low diffusion of colors or artefacts along the cellular boundary with improved sharpness compared to the noisy data. Notably, the nucleoporins (in green) surrounding the yeast nucleus are more clearly visible compared to the other techniques.

## Discussion

Imaging in fluorescence microscopy suffers from the drawback of measurement noise and photon noise, which severely degrades the resolution of the captured image. The objective of this paper is to address the issue of noise removal (denoising) in fluorescence microscopy. The proposed algorithm is a two stage process. In the first step, essential features of the image are retrieved, in the form of a mask (set ${ {\mathcal M} }_{\infty }$, discussed in Methods), with the corrupting noise eliminated almost completely. In the second stage, a noise free version of the image is reconstructed from the information of the gradients restricted to this mask. The gradients that correspond to the extracted features of the image are the most informative and least corrupted gradients of the image. Hence, when we try to reconstruct the image from these gradient information, noise propagation is minimum (and negligible in certain cases) and the result is a resolution enhanced denoised image. The proposed method is therefore based on a multifractal decomposition of the noisy image in which we isolate the most informative set ${ {\mathcal M} }_{\infty }$ as defined by equation 5. From this set a denoised image is computed with the help of a diffusion process, which is less sensitive to Poisson-Gaussian noise perturbations on the boundary set ${ {\mathcal M} }_{\infty }$. This explains the quality of results obtained w.r.t. the competing algorithms.

The merit of this algorithm can be seen from its denoising results on real fluorescence microscopy data, shown in Figs 5 and 6 as well as on simulated fluorescence microscopy data shown in Fig. 2. The competing algorithms are the state-of-the-art fluorescence microscopy image denoising algorithms, and are widely popular in the scientific community. Their performance when compared with the proposed scheme clearly shows the latter’s superiority. This is further validated through quantitative analysis, of all the denoising algorithms, on the simulated data as is shown in Fig. 3. The proposed method achieves the lowest residual error, which further validates its merit.

## Supplementary information

Supplementary Information
