# Supplementary material for: A Feature based Reconstruction Model for Fluorescence Microscopy Image Denoising
Source: Sci Rep. 2019 May 22;9:7725. doi: 10.1038/s41598-019-43973-2 (PMC6531475; doi:10.1038/s41598-019-43973-2)
Supplement: Supplementary file 1 — Supplementary Information [file 41598_2019_43973_MOESM1_ESM.pdf]

A Feature based Reconstruction Model for Fluorescence Microscopy Image Denoising

Suman Kumar Maji[1] **and Hussein Yahia**[2]

[1] Department of Computer Science & Engineering, Indian Institute of Technology Patna, Patna 800 013, India

[2] Team Geostat, INRIA Bordeaux Sud-Ouest, 200 rue de la Vieille Tour, 33405 Talence Cedex, France

[1]Email: smaji@iitp.ac.in

[2]Email: hussein.yahia@inria.fr

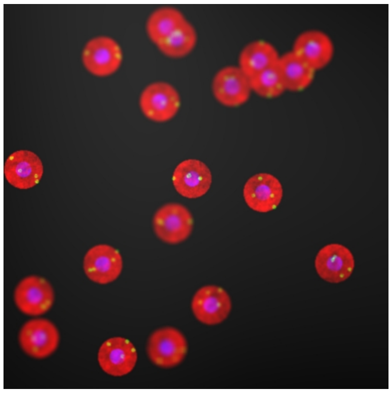

(a) Data 1

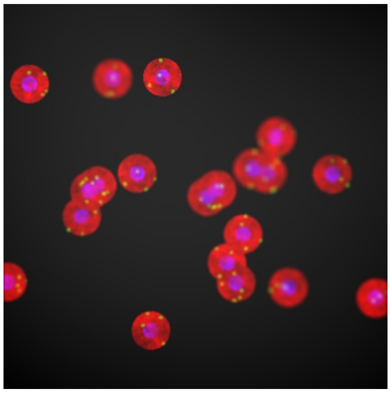

(b) Data 2

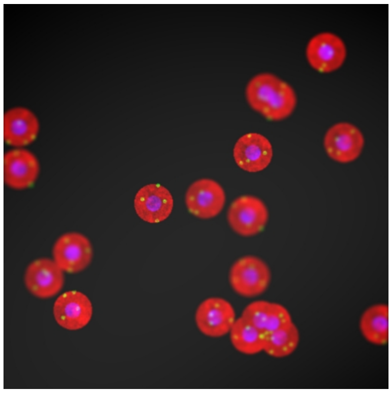

(c) Data 3

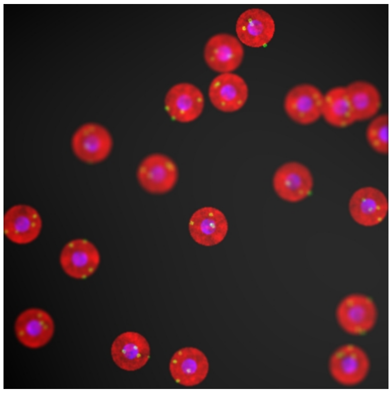

(d) Data 4

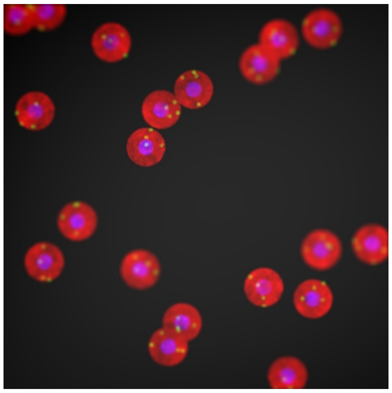

(e) Data 5

Fig. S1: **Simulated multichannel fluorescence microscopy data sets used for experiments.** Note: Figures and graphs corresponding to Data 1 are used in the manuscript for demonstration purpose. The results for the rest are shown in the Supplementary.

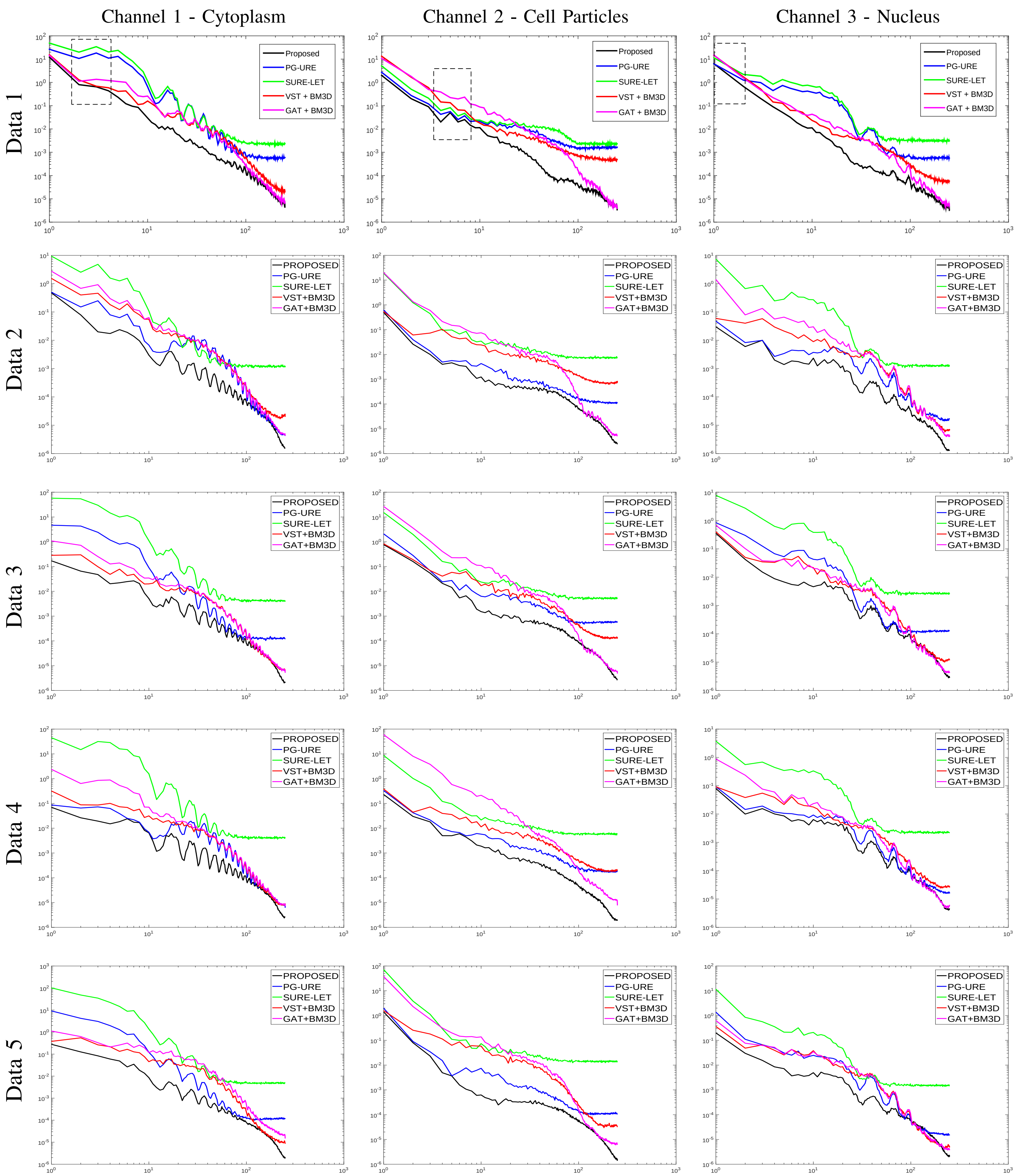

Fig. S2: **Quantitative analysis of denoising results over simulated fluorescence microscopy data set, in terms of residual power spectral density (PSD)**. The residual PSD's are computed as discussed in section **Results** of the manscript, and plotted against spatial frequency in the log-log scale.

TABLE S1: **Quantitative evaluation:** PSNR values for different noise levels.

		Noise Levels				
Data	Methods	10^{-3}	$10^{-2.5}$	10^{-2}	$10^{-1.5}$	10^{-1}
Data 1	PG - URE	32.43	32.02	30.64	26.64	22.17
	VST	28.14	26.81	27.88	18.90	19.70
	SURE	20.07	20.30	20.63	19.95	19.76
	GAT	24.61	23.88	23.37	21.43	19.88
	PROPOSED	**34.52**	**33.62**	**32.53**	**28.39**	**25.17**
Data 2	PG - URE	27.31	26.81	25.94	24.35	19.36
	VST	22.23	22.71	22.09	22.04	19.62
	SURE	18.21	18.59	18.03	17.07	16.05
	GAT	19.81	19.24	18.64	18.22	17.88
	PROPOSED	**29.59**	**29.51**	**29.02**	**27.17**	**23.72**
Data 3	PG - URE	30.17	29.59	29.14	24.59	19.81
	VST	24.68	24.70	24.32	22.44	21.52
	SURE	18.21	18.17	18.10	17.51	17.01
	GAT	22.84	21.2	20.88	18.81	16.44
	PROPOSED	**31.75**	**30.90**	**30.06**	**26.8**	**23.87**
Data 4	PG - URE	31.84	31.72	29.1	25.49	22.91
	VST	24.22	23.72	22.64	23.27	21.62
	SURE	16.09	15.88	16.07	15.35	15.08
	GAT	27.68	26.6	25.48	22.11	20.81
	PROPOSED	**33.39**	**32.26**	**30.39**	**26.66**	**23.41**
Data 5	PG - URE	31.47	29.51	28.73	23.88	22.89
	VST	27.38	25.86	24.71	23.62	22.46
	SURE	17.75	17.83	17.61	17.34	16.74
	GAT	28.16	25.58	25.23	21.97	18.74
	PROPOSED	**35.67**	**34.79**	**29.87**	**25.45**	**24.54**

TABLE S2: **Quantitative evaluation:** MSE values for different noise levels.

		Noise Levels				
Data	Methods	10^{-3}	$10^{-2.5}$	10^{-2}	$10^{-1.5}$	10^{-1}
Data 1	PG - URE	0.0006	0.0007	0.0008	0.002	0.003
	VST	0.0015	0.002	0.004	0.0107	0.012
	SURE	0.0090	0.0093	0.0098	0.0101	0.0105
	GAT	0.0046	0.0048	0.005	0.008	0.01
	PROPOSED	**0.0004**	**0.00044**	**0.0005**	**0.001**	**0.0014**
Data 2	PG - URE	0.002	0.003	0.0044	0.0068	0.012
	VST	0.005	0.006	0.0066	0.0072	0.0108
	SURE	0.014	0.015	0.0156	0.03	0.08
	GAT	0.01	0.011	0.014	0.018	0.02
	PROPOSED	**0.001**	**0.0016**	**0.002**	**0.0028**	**0.004**
Data 3	PG - URE	0.0009	0.001	0.003	0.0035	0.0101
	VST	0.0034	0.0041	0.005	0.0056	0.007
	SURE	0.0152	0.0154	0.0161	0.0177	0.0198
	GAT	0.0052	0.008	0.01	0.04	0.08
	PROPOSED	**0.0006**	**0.0007**	**0.0009**	**0.0021**	**0.0041**
Data 4	PG - URE	0.0006	0.00065	0.0012	0.005	0.009
	VST	0.0037	0.0042	0.0054	0.007	0.09
	SURE	0.0246	0.0258	0.036	0.046	0.08
	GAT	0.0017	0.002	0.0026	0.005	0.01
	PROPOSED	**0.00045**	**0.0005**	**0.0006**	**0.0008**	**0.001**
Data 5	PG - URE	0.0007	0.0011	0.0013	0.004	0.0045
	VST	0.0018	0.0025	0.0034	0.0043	0.0049
	SURE	0.0164	0.0167	0.0173	0.0184	0.0211
	GAT	0.0015	0.0027	0.0029	0.0063	0.0133
	PROPOSED	**0.00027**	**0.00032**	**0.001**	**0.0028**	**0.0034**

TABLE S3: **Time cost comparison (in seconds).**

	Methods				
	PG-URE	VST+BM3D	GAT+BM3D	SURE-LET	Proposed
Data 1	2.07	2.045	2.33	0.808	1.88
Data 2	1.65	2.29	2.58	0.35	1.30
Data 3	2.17	2.38	2.76	0.44	1.89
Data 4	1.80	2.19	2.41	0.32	1.49
Data 5	1.94	2.14	2.45	0.34	1.46
Experiment 391	1.44	2.36	2.48	0.42	1.06
Experiment 617	1.43	2.16	2.54	0.52	1.10
